# Structural Study on Fat Crystallization Process Heterogeneously Induced by Graphite Surfaces

**DOI:** 10.3390/molecules25204786

**Published:** 2020-10-19

**Authors:** Fumitoshi Kaneko, Yoshinori Yamamoto, Shinichi Yoshikawa

**Affiliations:** 1 Graduate School of Science, Osaka University, Toyonaka 560-0044, Japan; yamamotoy16@chem.sci.osaka-u.ac.jp; 2 Research Institute for Creating the Future, Fuji Oil Holdings Inc., Izumisano 598-8540, Japan; yoshikawa.shinichi@so.fujioil.co.jp

**Keywords:** fat crystallization, heterogeneous nucleation, crystal polymorph, molecular orientation, trilaurin, graphite

## Abstract

Some inorganic and organic crystals have been recently found to promote fat crystallization in thermodynamically stable polymorphs, though they lack long hydrocarbon chains. The novel promoters are talc, carbon nanotube, graphite, theobromine, ellagic acid dihydrate, and terephthalic acid, among which graphite surpasses the others in the promotion effect. To elucidate the mechanism, we investigated the influence of graphite surfaces on the crystallization manner of trilaurin in terms of crystal morphology, molecular orientation, and crystallographic features. Polarized optical microscopy, cryo-scanning electron microscopy, synchrotron X-ray diffractometry, and polarized Fourier-transform infrared spectroscopy combined with the attenuated total reflection sampling method were employed for the analyses. All the results suggested that the carbon hexagonal network plane of graphite surfaces have a high potential to facilitate the clustering of fat molecules against high thermal fluctuations in fat melt, the fat molecules form a layer structure parallel to the graphite surface, and the clusters tend to grow into thin plate crystals of the β phase at the temperatures corresponding to low supercooling. The β′ phase also has a larger chance to grow on the graphite surface as supercooling increases.

## 1. Introduction

Surfactants and materials having long hydrocarbon chains have been used as additives to promote fat crystallization [1]. The alkyl chains of these compounds are considered to act as templates for the heterogeneous nucleation by interacting with the hydrocarbon-chain moieties of fat molecules and supporting their ordering processes [2,3]. In contrast, recent studies found that some inorganic and organic crystals (i.e., talc, carbon nanotube, graphite, theobromine, ellagic acid dihydrate, and terephthalic acid) promoted fat crystallization in thermodynamically stable polymorphs [4,5]. These additives are sparingly soluble in lipids and possess no long hydrocarbon chains in their chemical structure, which implies that the additive surfaces attract fat molecules strongly and urge them to form crystal nuclei.

The novel promoters have a great potential to be applied to the production and separation processes in the oleochemical industry. Information about the structural relationship between the surface of the novel promoters and the heterogeneously nucleating fat crystals thereon, as well as the resultant changes in the fat crystallization process, is crucial to understand the role of the novel promoters and to develop more effective promoters.

Triacylglycerols (TAGs) are the main components of natural lipids, such as vegetable oil and animal fats, and exhibit polymorphism of crystals [6,7]. Since TAGs are essential ingredients in a wide variety of industrial products and their polymorphism and solid-state structures often exert a significant influence on the quality and properties of the final products, we have been particularly interested in how the novel promoters interact with TAG molecules, and how the crystallization behavior of TAGs changes under their influence.

In this study, we have investigated the influence of graphite surfaces on the crystallization manner of trilaurin (LLL), since graphite powder promoted crystallization of LLL more effectively than the other new promoters [4]. LLL is a saturated monoacid TAG having three dodecanoic acid molecules ester-bonded to one glycerol. LLL shows three crystal polymorphs of the α, β′ and β phases, differing in thermodynamic stability and crystal structure [8]. Melting points and structural features of LLL polymorphs are summarized in Table 1.

Graphite features a stacked structure of carbon hexagonal-network planes [9], and therefore, the graphite surface with the network would play a crucial role in the promotion effects on fat crystallization. To tackle this issue, we have conducted an elaborate examination on the crystallization of LLL at surfaces of highly oriented pyrolytic graphite (HOPG). Furthermore, to approach this issue from different points of view, we have employed the following methodologies: polarized optical microscopy (POM), cryo-scanning electron microscopy (Cryo-SEM), synchrotron X-ray diffractometry (SR-XRD), and polarized Fourier-transform infrared spectroscopy combined with the attenuated total reflection sampling method (polarized FTIR ATR).

Morphological observations using POM and Cryo-SEM provide information about how the growth behavior of LLL crystals change with the crystallization conditions at the HOPG surfaces. SR-XRD, which combines a highly bright X-ray source and high-sensitively detectors, allows us to detect microstructural changes during the crystallization process of LLL [10,11,12]. Furthermore, polarized FTIR ATR helps us to analyze the molecular-level structure and orientation of LLL crystals grown on the HOPG surfaces.

In this paper, we will describe the characteristics of fat crystallization induced by the HOPG surfaces based on the experimental data acquired, and discuss how graphite effectively facilitates the heterogeneous nucleation of fat crystals in the stable polymorphs at the surfaces with carbon hexagonal networks.

## 2. Results and Discussion

### 2.1. Microscopic Observation of LLL Crystals on HOPG Sheets

We have followed the morphological change of LLL crystals growing on the HOPG surface using POM. Two kinds of melt-crystallization procedures with constant-rate cooling or isothermal cooling were employed. The POM images acquired clearly displayed that the morphology of LLL crystals significantly depends on the crystallization conditions.

#### 2.1.1. Melt-Crystallization with Constant-Rate Cooling

**Figure 1:**
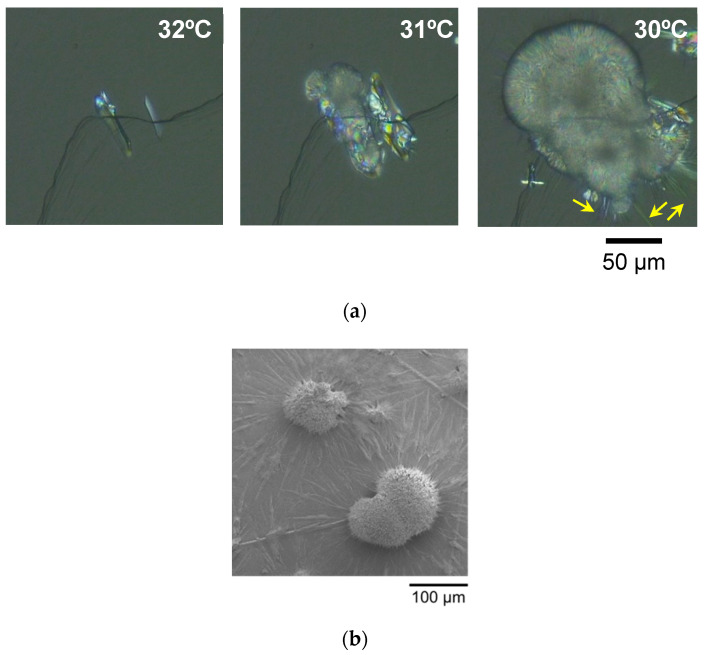

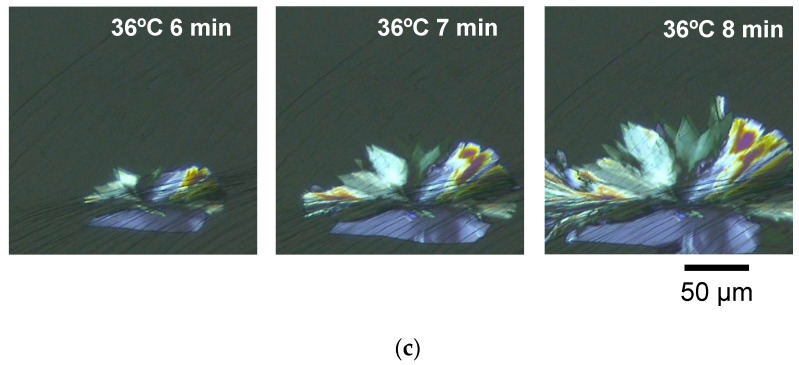
Morphology of LLL crystals growing on highly oriented pyrolytic graphite (HOPG) sheets. (**a**) Polarized optical microscope (POM) images taken in the melt-crystallization process with a constant cooling rate of 1 °C/min, (**b**) a cryo-scanning electron microscope (Cryo-SEM) image taken after the stabilization process, and (**c**) POM images taken in the melt-crystallization process with isothermal cooling at 36 °C.

Figure 1a shows POM images of LLL crystals developing on the HOPG sheet in the melt-crystallization process with a constant cooling rate of 1 °C/min. In this figure, dark lines running diagonally correspond to layered steps of the HOPG surface, which were made in preparing the HOPG sheet by cleavage before the observation. At 32 °C, LLL crystals growing in a thin plate shape were observed in the vicinity of the steps. With further cooling, newly occurring crystals grew radially from the edges of the plate-shaped crystals to form globular crystals (31 °C and 30 °C). The globular crystals grew fast, then finally overwhelmed the previously occurring plate-shaped crystals; meanwhile, needle-shaped crystals protruded from the peripheries of the globular crystals, as indicated by arrows in Figure 1a.

The same morphologies of LLL crystals were confirmed also by the Cryo-SEM observation. Figure 1b shows a Cryo-SEM image of the grown LLL crystals on the HOPG surface, which was taken after the cooling to 0 °C and the subsequent stabilization process with heating at 35 °C for 10 min followed by cooling down to 20 °C. The globular crystals with a diameter of ~100 μm sat on the HOPG surface. The needle-shaped crystals streaked along the HOPG surface and seemed to have radiated out from the centers locating under the globular crystals. The plate-shaped crystals were not found, probably because of being covered completely by the globular crystals.

#### 2.1.2. Melt-Crystallization with Isothermal Cooling

Figure 1c shows POM images of LLL crystals developing on the HOPG sheet in the melt-crystallization process with isothermal cooling at 36 °C. When the cooling time passed ~4 min, flake-shaped crystals occurred first in the vicinity of layered steps of the HOPG surface. These crystals grew away from the steps along the smooth surface of HOPG and, shortly afterward, dendritic crystals grew from the peripheries to disturb the growth direction of the flake-shaped crystals. However, no globular crystals were observed in this isothermal condition.

### 2.2. Structural Changes during Crystallization

The above microscopic observation suggests that steps of the HOPG surface acted as the heterogeneous nucleation sites for LLL crystals and that at least two kinds of crystal polymorphs were involved in the crystallization of LLL on HOPG sheets. To study the polymorphic phase transition of LLL crystals occurring in the different crystallization conditions, we conducted time-resolved SR-XRD measurements using neat LLL and LLL in contact with a HOPG sheet (LLL/HOPG).

#### 2.2.1. Crystallization of Neat LLL with Constant-Rate Cooling and Heating

**Figure 2:**
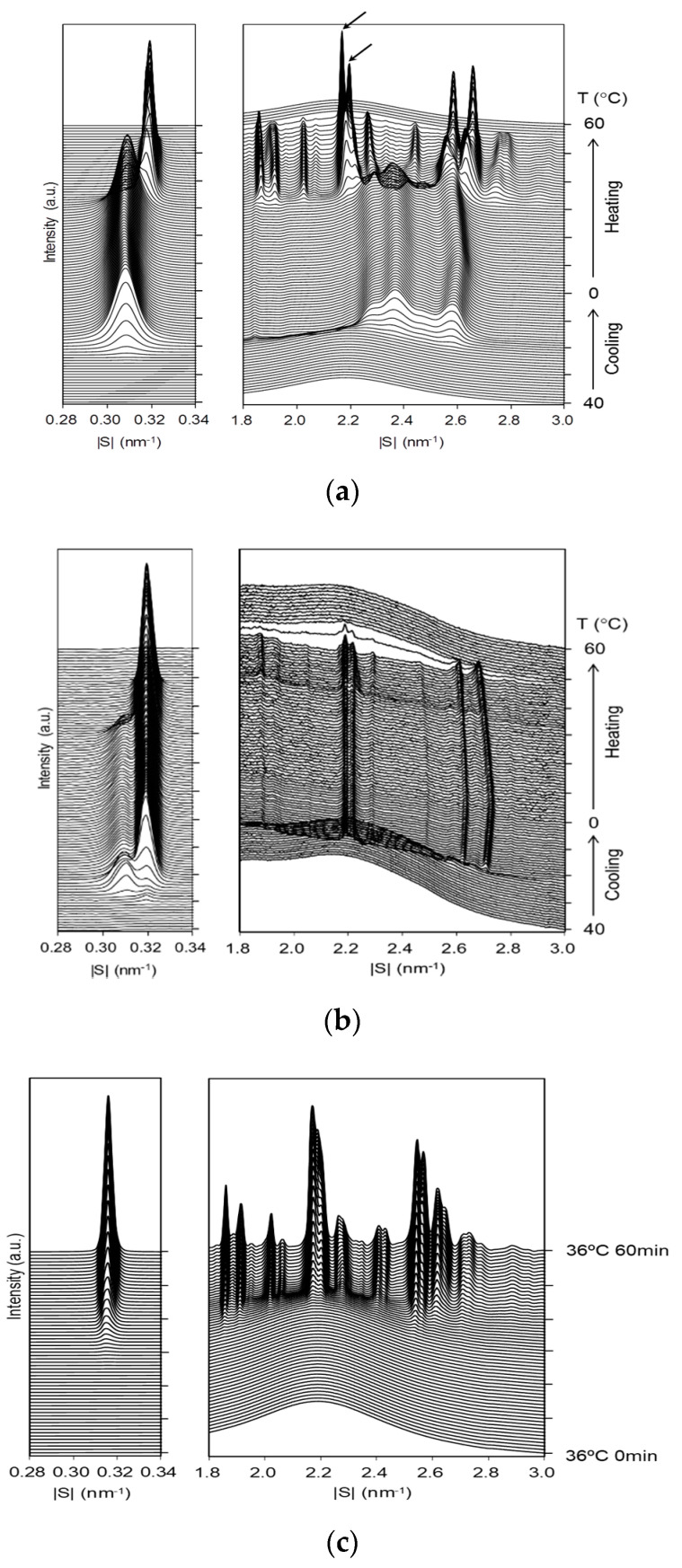
Time-dependent changes of synchrotron X-ray diffraction (SR-XRD) profiles in small-angle X-ray scattering (SAXS) and wide-angle X-ray scattering (WAXS) regions. (**a**) Crystallization of neat LLL with a constant cooling rate of 1 °C/min, (**b**) crystallization of LLL/HOPG with the same cooling rate, and (**c**) isothermal crystallization at 36 °C of LLL/HOPG.

Figure 2a shows the SR-XRD profiles of neat LLL during crystallization with a constant cooling rate of 1 °C/min and the subsequent heating at a rate of 5 °C/min. The crystallization started at about 23 °C with the occurrence of the β′ phase, which is identified from a small-angle X-ray scattering (SAXS) peak at a scattering vector |*s*| = 0.309 nm^−1^ (lattice spacing *d* = 3.24 nm) and wide-angle X-ray scattering (WAXS) peaks at |*s*| = 2.37 and 2.58 nm^−1^ (*d* = 0.423 nm and 0.387 nm). No essential changes took place during the further cooling. In the heating process, these β′ peaks disappeared at ~30 °C and the peaks of the β phase characterized by the following peaks appeared instead: a SAXS peak at |*s*| = 0.318 nm^−1^ (*d* = 3.15 nm) and major WAXS peaks at |*s*| = 2.18, 2.56, and 2.63 nm^−1^ (*d* = 0.459, 0.390, and 0.380 nm). Based on the results of our previous study with DSC, POM, and XRD [4], this phenomenon is attributed to the polymorphic phase transition from the meta-stable β′ phase to the stable β phase. Further heating caused only minor changes in the SAXS and WAXS profiles: the SAXS peak slightly shifted to the wider-angle direction at ~40 °C, and the main WAXS peak at |*s*| = 2.18 nm^−1^ (*d* = 4.59 nm) split into two peaks at |*s*| = 2.17 and 2.19 nm^−1^ (*d* = 4.61 and 4.57 nm) as pointed out by the arrows in Figure 2a.

To summarize, neat LLL melt crystallized in the β′ phase in the cooling at a constant rate, and the β phase emerged only in the subsequent heating through the phase transition from the β′ phase.

#### 2.2.2. Crystallization of LLL/HOPG with Cooling and Heating at Constant Rates

Figure 2b shows the SR-XRD profiles of LLL/HOPG during crystallization with a constant cooling rate of 1 °C/min and the subsequent heating at a rate of 5 °C/min. In the cooling process, a SAXS peak at |*s*| = 0.319 nm^−1^ (*d* = 3.13 nm) appeared first at ~30 °C and grew slowly during cooling from 30 to 20 °C. Immediately after this, another SAXS peak at |*s*| = 0.309 nm^−1^ (*d* = 0.324 nm) appeared at ~26 °C, increased its intensity during cooling from 26 to 23 °C, then attenuated to a certain level. These changes in the SAXS profile indicate the sequential crystallization events of LLL/HOPG as follows: crystallization in the stable β phase, crystallization in the metastable β′, and partial phase transition from β′ to β. With further cooling, the SAXS profile did not change any more. In the heating process, the peak of the β phase increased its intensity again at the expense of the peak of the β′ phase, which started at ~30 °C. The peak of the β′ phase disappeared at ~33 °C, and then that of the β phase disappeared completely at ~50 °C.

The WAXS profile showed corresponding changes, though the occurrence of the β phase in the cooling was not detected because of its small intensity. In the cooling process, the peak at |*s*| = 2.58 nm^−1^ (*d* = 0.391 nm) for the β′ phase appeared at ~25 °C, and the peaks at |*s*| = 2.19, 2.21, 2.61, and 2.69 nm^−1^ (*d* = 0.457, 0.452, 0.383, and 0.372 nm) for the β phase appeared at ~23 °C. In the subsequent heating process, the peaks of the β phase disappeared at ~50 °C after the peak of the β′ phase faded.

A comparison of the SR-XRD profiles between neat LLL and LLL/HOPG shows that the HOPG surface has the following influence on crystallization of LLL: (1) The HOPG surface preferentially promoted crystallization of LLL in the β phase, so that the β phase appeared ahead of the β′ phase, and (2) crystallization of LLL in the β′ phase was promoted to a small extent, indicating the direct or indirect interactions with the HOPG surface.

#### 2.2.3. Crystallization of LLL/HOPG with Isothermal Cooling

Figure 2c shows the SR-XRD profiles of LLL/HOPG during crystallization with the isothermal cooling at 36 °C for 60 min. No peaks due to crystalline phases appeared for about half an hour, both in the SAXS and WAXS regions. After ~35 min, the peaks due to the β phase appeared; specifically, a SAXS peak at |*s*| = 0.315 nm^−1^ (*d* = 3.17 nm) and WAXS peaks at |*s*| = 2.17, 2.21, 2.55, and 2.57 nm^−1^ (*d* = 0.461, 0.452, 0.392, and 0.389 nm, respectively). These peaks gradually increased in intensity over time. No peaks due to the β′ phase appeared at this temperature.

#### 2.2.4. Structural Changes of LLL Crystals on a HOPG Sheet

By combining the information obtained by the morphological observation and the SR-XRD measurements, the following conclusions can be drawn about the crystallization process of LLL on a HOPG sheet:

1. A neat LLL melt tends to crystallize into the β′ phase in the constant-rate cooling. However, the β phase appears on the hexagon network of graphite as thin plate crystals in the constant-rate cooling. Since the growth rate of the β phase is low, the surrounding melt keeps its liquid state during further cooling.

2. When the supercooling of the remaining LLL melt reaches to a certain level, the β′ phase occurs as globular crystals on the thin plate crystals of the β phase. The globular crystals grow faster than the plate crystals of the β phase. The temperature for the β′ phase to appear is clearly higher than that in the crystallization of neat LLL. The crystal face of β would support the nucleation of the β′ phase.

3. The β phase is thermodynamically more stable than the β′ phase, and therefore, the β′ → β solid-state phase transition takes place spontaneously when thermally activated, as can be seen in the heating process of neat LLL.

4. Within the globular crystals on the HOPG sheet, the β′ → β phase transition proceeds, since they start to grow around 30 °C. It is a striking contrast to the β′ phase generated in the neat LLL melt; the β′ phase, which appears around 23 °C, practically has no chance to start the solid-state transition.

There are some differences in timing for structural changes between morphological observation and XRD experiments. We will deal with this issue in a later section.

### 2.3. Polymorph and Orientation of LLL Crystal on a Graphite Sheet

As described above, thin plate crystals of LLL in the β phase start to grow on the HOPG sheet. Similarly, plate crystals of LLL in the β phase can be obtained also by solution crystallization. Since the flat surface of the solution-grown crystal corresponds to the lamellar plane covered with methyl groups, it can be inferred that the carbon hexagon network of graphite facilitates the formation of the double-chain layer structure parallel to it.

**Figure 3:**
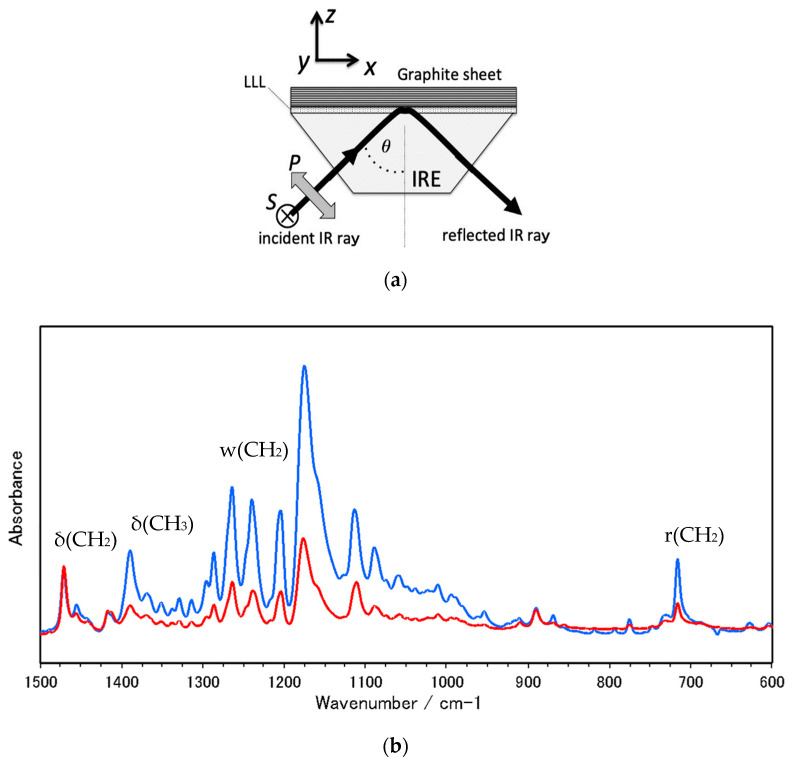

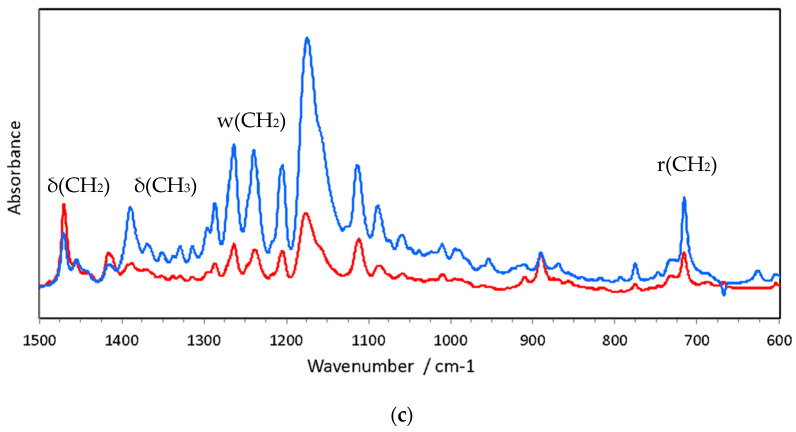
Polarized attenuated total reflection Fourier-transform infrared (ATR-FTIR) spectra of LLL β-form crystals with blue and red lines for p and s polarizations: (**a**) experimental arrangement, (**b**) spectra taken for LLL/graphite sheet, and (**c**) spectra taken for solution-grown single crystals, whose flat faces are set parallel to the internal reflection element (IRE) sampling face.

To confirm this expectation, we have studied the crystalline state of LLL generated on a graphite sheet by polarized FTIR ATR method [13], which can probe organic layers on an opaque substrate efficiently. Figure 3a shows the experimental conditions used and Figure 3b shows the spectra taken for LLL crystal generated on a graphite sheet. The characteristics of polarized ATR spectroscopy are summarized in Appendix A.

The LLL layer generated on a graphite sheet shows the characteristics of an ordered solid phase of TAGs. The pronounced IR bands can be assigned to the vibrational modes of lauroyl chains of LLL [14,15,16,17]. A band at 717 cm^−1^ and a band at 1472 cm^−1^ are due to CH_2_ rocking, r(CH_2_), and CH_2_ scissoring, δ(CH_2_), respectively. A series of bands in the 1350−1180 cm^−1^ region are attributed to the progression bands of CH_2_ wagging, w(CH_2_), modes. The band at 1380 cm^−1^ is assigned to the methyl symmetric deformation, δ_s_(CH_3_). The r(CH_2_) and δ(CH_2_) bands are sensitive to the lateral packing of hydrocarbon chains. Their frequencies and appearance as sharp singlets strongly suggest that the acyl chains form the T// sub-cell structure, which is characteristic of the β phase. Furthermore, the appearance of the w(CH_2_) progression bands indicates the high conformational regularity of lauroyl chains. Accordingly, the LLL crystal layer on the graphite sheet can be regarded as the stable solid phase β.

The LLL crystal layer shows a clear polarization dependence. The most marked intensity difference between p and s polarizations is seen in the w(CH_2_) progression bands, and the δ(CH_3_) band, whose transition moments are roughly parallel to the chain axis, appear significantly more intense in p-polarization than in s-polarization for the two kinds of LLL samples. The r(CH_2_) band, whose transition moment directs perpendicular to the skeletal plane, also appears more intense in p-polarization. On the other hand, there is no clear difference in intensity between p- and s-polarization for the δ(CH_2_) band, whose transition moment is in the skeletal plane and directs to the bisector of the C-C-C angle.

**Figure 4:**
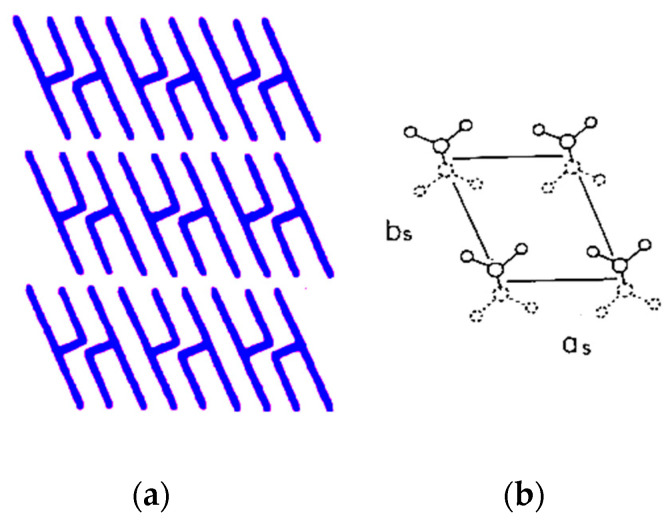
Schematic representation for the β phase of monoacid triacyl glycerols (TAGs). (**a**) Molecules form a double-chain-layered structure, whose interface is parallel to the flat surface of thin plate single crystals. (**b**) Acyl chains in the β phase form the T// sub-cell structure.

The polarization of these bands strongly suggests that the lamellae of the β phase are set parallel to the surface of the graphite sheet. The β phase of monoacid TAGs forms a layer structure of double-chain length, as depicted in Figure 4a [18,19,20,21]. The lamellar interface consists of the methyl terminals of acyl chains that incline by about 30° from the normal of the interface, principally toward the a_s_ axis of the T// sub-cell shown in Figure 4b. When the lamellar interface is set parallel to the IRE sampling face, the transition moments of w(CH_2_), δ(CH_3_), and r(CH_2_) modes possess a considerable amount of z-component, leading to their more prominent absorption in p-polarization, as actually observed.

Polarized FTIR ATR measurements on solution-grown plate crystals of the β phase obviously support the above results. The polarized spectra exhibit the same characteristics both in frequencies and polarization, as shown in Figure 3c. These observations clearly suggest that the graphite surface with a carbon hexagon network urges LLL melt to form the β phase with a lamella parallel to it.

## 3. Discussion

### 3.1. Roles of Graphite Surfaces

As described above, the experiments conducted in this study using HOPG sheets have brought some information on the roles of the graphite surface. The morphological observation shows that the fat crystallization starts on the graphite surface with carbon hexagon network forming thin plate crystals, which are confirmed to be the β phase through X-ray and ATR FTIR measurements. Furthermore, the polarized ATR FTIR spectra indicate that the double-chain molecular layers of the thin plate crystal are parallel to the graphite surface. The molecular orientation seems to be reasonable since generally, chain molecules tend to form plate crystals elongated in the lateral direction of hydrocarbon chains.

These experimental results clearly indicate that the carbon hexagonal network of graphite provides suitable sites for the nucleation of the β phase. The graphite surface would reduce the surface energy of the β phase, and therefore, the activation energy for the nucleation would decrease markedly, resulting in the occurrence of the β phase on the graphite surface.

Without using this additive effect, the generation of the β phase directly from a neat melt is highly difficult; as show in Figure 2a, an ordinary continuous cooling procedure only leads to the occurrence of the β′ phase, except for very slow cooling. According to the ordinary view of crystal growth [22,23,24], TAG molecules are considered to form embryos, precursory clustered molecules for crystal nuclei: the frequency of occurrence for the embryos of each polymorph would increase with supercooling. Since the supercooling, namely the driving force for crystallization, is always advantageous to the β phase, the dominant occurrence of the β′ phase suggests that the β phase has a larger surface energy, and this acts against the β phase in the nucleation in ordinary cooling [25].

The graphite surface enables the β phase to appear at higher temperatures; as shown in Figure 2c, the graphite surface induces the isothermal crystallization of the β phase even at 36 °C. These observations suggest that the surface energy of a precursory cluster is significantly reduced when it is generated on the graphite surface, and the cluster manages to surpass the activation energy for nucleation to grow as the β phase by using this benefit.

Generally, crystal faces have many steps, as do the graphite faces. Such steps would interact with clustered molecules from lateral directions. If the interactions are favorable for the clustered molecules, the steps on the graphite surface would greatly help the nucleation of fat molecules by reducing the surface energy. This effect would make it easier for TAG molecules to form clusters on the graphite surface, especially in the early stage of clustering. The impact of the steps in the new additives is one of the important research topics for the future.

### 3.2. Occurrence of the β′ Phase 

At the moment, the influence of the graphite surface on the occurrence of the β′ phase is uncertain. The present study confirms the occurrence of the β′ phase at higher temperatures under the influence of HOPG surfaces. However, the β′ phase occurs only after the β phase is generated with the help of graphite surfaces, and it is possible that the β phase promotes the development of the β′ phase. In fact, the microscopic observation shows that the globular crystals of the β′ phase are sitting on the surface of the plate crystals of the β phase, suggesting another possibility that the crystal face of the β phase facilitates the nucleation of the β′ phase from a supercooled melt. We presently have not enough experimental evidence to judge whether the graphite surface or the previously generated β plate crystal’s surface promotes the nucleation of the β′ phase. In either case, the occurrence at a higher temperature has an influence on the subsequent β′ → β solid-state phase transition.

When generated at a higher temperature, the β′ phase would be thermally agitated to transform to the more stable β phase. This speculation can help to explain why both the crystal growth of the β phase and the β′ → β phase transition take place in the cooling process of LLL/HOPG and why the β′ phase does not transform in the cooling process of neat LLL. 

### 3.3. Later Stages of Crystallization

After the nucleation on the graphite surface, the β phase grows as thin plate crystals for a while, keeping its flat face parallel to the graphite surface. However, during its growth in a supercooled melt, the morphology and arrangement of the plate crystals gradually become disordered, leading to crystal aggregation and deformation. As for the β′ phase, it grows as globular crystals from the initial stage of its crystallization, at least at the optical microscopic level.

**Figure 5:**
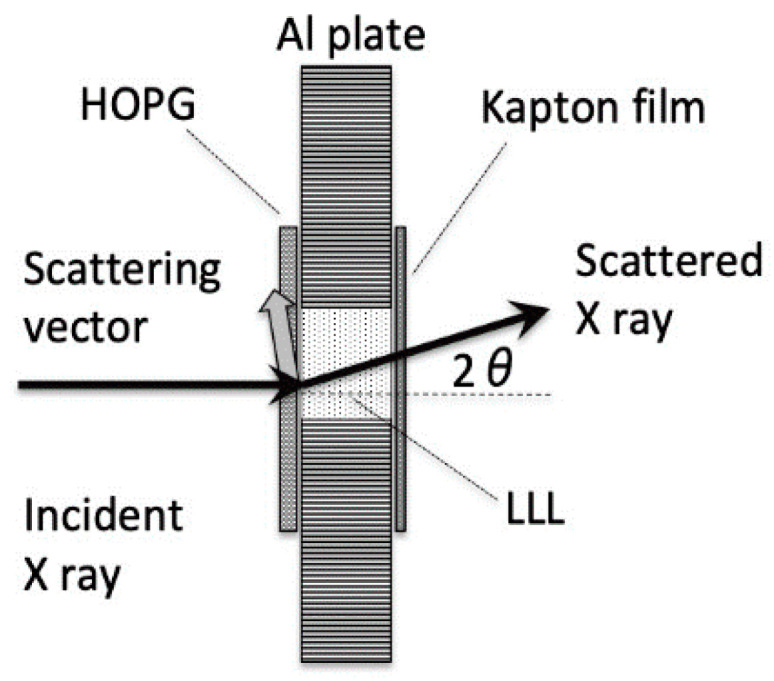
Experimental arrangement of LLL/HOPG in SR-XRD.

The growth behavior of the β and β′ phases is considered to have a crucial influence on the time-dependent changes observed by the SR-XRD method, such as, the intensities of reflections and the timing for each event in the system. Figure 5 shows the sample cell and its experimental arrangements. The incident X-ray radiation impinges the HOPG sheet normally, and the scattering vector can only point in a limited range of directions, mostly, nearly parallel to the HOPG surface. Accordingly, only a few lattice points can exhibit X-ray reflections. When the plate crystals of the β phase are set parallel to the HOPG plane, there is no chance for the reflections characteristic of the β phase to appear. These reflections would be able to appear only after the β phase forms crystal aggregation with disordered arrangement. On the other hand, the β′ phase forming globular crystals would tend to appear from the early stage of crystal growth. Another problem of SR-XRD experiments is that the size of the X-ray beam (about 0.2 × 0.2 mm) is not large enough to cover all the structural changes occurring in the sample cell with a diameter of 1.5 mm. 

It can be assumed that the combined effect of the growth behavior and the X-ray experimental conditions leads to the following phenomena we observe in the experiments using the LLL/HOPG sample:

- In the cooling at a rate of 1 °C/min, the occurrence of the β phase was observed by SAXS at 30 °C, while at 32 °C by microscopic observation.
- As for the isothermal crystallization at 36 °C, the occurrence of the β phase was confirmed by microscopic observation after holding only for several minutes, while the reflections due to the β phase finally appeared after about half an hour in the SR-XRD measurements.
- In the continuous cooling crystallization, the reflections of the β phase were weak compared with those of the neat LLL sample.

Although it is difficult to accurately assess the magnitude of the above-mentioned effects resulting from the experimental conditions and the growth behavior, it is assumed that the experimental results obtained by SR-XRD would be affected more or less by the time-dependent orientational and morphological changes of growing crystals.

In relation to this issue, we would like to make a comment on our previous study on the new additive effect. At that time, we observed that only weak reflections due to long spacings appeared during the crystallization process promoted by a powder of graphite, and speculated that the crystallization started from the clusters of LLL molecules with acyl chains adhered in parallel to the carbon hexagon network [4]. However, we have recently noticed that crystalline regions growing from graphite particles interfere with each other when they come into contact, and as a result, some preferential orientations of crystal region are generated in the specimen. Some resultant crystalline samples have showed quite weak long-space reflections, as we observed previously. Through these experiences, we have learned that the inhomogeneous orientation of crystalline tissues would happen even in the heterogeneous crystallization induced by fine powder additives if certain conditions are met.

## 4. Materials and Methods

### 4.1. Samples

LLL with ≥99% purity was purchased from Sigma-Aldrich (St. Louis, USA) and used without further purification. Graphite sheets of Grafoil® GTA with 0.076 mm thickness were purchased from NeoGraf (Lakewood, CA, USA). HOPG of SPI® HOPG GRADE 1 was purchased from Structure Probe (West Chester, PA, USA) and cleaved to a thin layer for usage. 

### 4.2. Polarized FTIR Spectroscopy with ATR Sampling Technique

LLL was crystallized on the graphite sheet by heating at 80 °C for 10 min and subsequently cooling down to 25 °C at a rate of 1 °C/min. For comparison, platelet single crystals of LLL in the β phase were grown from a hexane solution. The flat face of grown single crystals corresponds to the (001) plane, i.e., the lamellae of LLL are stacked along the normal of this face [8].

For polarized FTIR spectroscopy, a PerkinElmer Spectrum Two FTIR spectrometer (PerkinElmer, Inc., Waltham, MA, USA), a Czitek Microm ATR accessory (Czitek, Danbury, CT, USA), and a JASCO PL81 wire-grid polarizer(JASCO Co., Hachioji, Japan) were employed. The ATR accessory was equipped with an internal reflection element (IRE) of diamond, which gave a refractive index of 2.38 in the region of 4000–400 cm^−1^. The measurements were conducted with an incident angle of 45°, a resolution of 2 cm^−1^, and a scan number greater than 8.

### 4.3. SR-XRD Analysis

Two kinds of specimens were prepared: a neat LLL melt and a LLL melt in contact with a HOPG sheet. For the neat LLL system, a hole (2 mmϕ) drilled in an aluminum plate with 1.5 mm thickness was filled with LLL, and then both sides of the hole were sealed with windows of polyimide (Kapton) films. For the LLL/HOPG system, the HOPG layer was placed inside the incident windows to be in contact with LLL, as illustrated in Figure 1.

SR-XRD measurements were carried out at BL-10C of Photon Factory, a synchrotron radiation facility of KEK (Tsukuba, Japan), by applying X-rays with a wavelength of 0.125 nm [26]. SAXS and WAXS from samples were simultaneously detected to obtain the information about long- and short-spacings of highly ordered anisotropic crystal lattices. Dectris PILATUS3 2M and PILATUS3 200K were employed as the SAXS and WAXS detectors, respectively. During the measurements, each sample was heated at 80 °C for 10 min, subsequently cooled down to 0 °C at a rate of 1 °C/min, and then reheated up to 80 °C at a rate of 5 °C/min. For LLL/HOPG, the SR-XRD profile was also taken under an isothermal condition by cooling at 36 °C for 60 min after quench cooling at a rate of 20 °C/min from 80 °C. The thermal control was done by using a thermoelectric cooling/heating device, Linkam 10002L. The cooling and heating rates were chosen to be the same as those in our previous study [4]. The relatively slow cooling rate is crucial; if it is too high, the effect of the additives is less likely to be reflected in polymorphism.

### 4.4. SEM Observation

Prior to being introduced to the Cryo-SEM unit, thermodynamically stabilized LLL crystals were prepared for LLL/HOPG by crystallizing LLL on an HOPG layer-attached aluminum pan. Using a cooling/heating device, Linkam 10021, crystallization was performed as follows: (i) heating at 80 °C for 10 min, (ii) cooling down to 0 °C at a rate of 1 °C/min, (iii) reheating up to 35 °C at a rate of 5 °C/min and holding at the temperature for 10 min, and (iv) re-cooling down to 20 °C at a rate of 1 °C/min. The procedures (iii) and (iv) were conducted to make the meta stable β’ phase transform to the stable β phase and to carry out SEM measurements at a temperature sufficiently lower than the melting point of the β phase.

The sample pan thus obtained was fixed onto a brass holder via a carbon seal binder and then transferred to the evacuated and pre-cooled sample chamber of a Gatan Alto 1000 cryo-preparation and coating station. In the cryo-chamber, each sample was coated with fine gold particles by means of ion-sputtering vapor deposition. Subsequently, the sample holder was directly introduced to the cold stage of a Hitachi High-Technologies SU3500 SEM equipped with an electron gun of a tungsten hairpin filament. SEM images of the sample surfaces were acquired as secondary electron images with a magnification of ×100 in a high vacuum mode (60 Pa) where electron beams with an acceleration voltage at 3 kV were applied to the sample cooled at −125 °C.

### 4.5. POM Observation

In contrast to the SEM imaging for the annealed LLL crystals, the morphology of LLL crystals changing with the crystal growth was observed by POM using a Keyence VHX-600 digital microscope. For neat LLL and LLL/HOPG, sample preparation and POM observation were carried out in the procedure as follows: (i) The melts of 4 μL LLL were placed on a glass plate and an HOPG-layer-attached glass plate, respectively. (ii) The samples were covered with cover slips, then heated at 80 °C for 10 min. (iii) LLL was crystallized by cooling at a rate of 1 °C/min or by isothermally cooled at 36 °C after quench cooling at a rate of 20 °C/min. (iv) During the crystallization processes, POM images were periodically taken with a magnification of ×500 in a reflection mode. The temperature of the samples was controlled by using a Linkam 10002L attached to a stage of the digital microscope.

## 5. Conclusions

We have studied the crystallization behavior of LLL induced by highly oriented graphite by employing POM, Cryo-SEM, SR-XRD, and polarized FTIR-ATR spectroscopy. The results indicate that the graphite surface with carbon hexagon network promotes the occurrence of the β phase through the heterogeneous nucleation, which starts with the formation of LLL clusters with double-chain length molecular layers oriented in parallel to the graphite surface.

## Figures and Tables

**Table 1:** Melting points and structural features of trilaurin (LLL) crystals.

	Previous Study [8]			Present Study ^1^		
Polymorphs	α	β′	β	β′ (0 °C)	β (36 °C)	β (50 °C)
Sub-cell Structure	H	O⊥	T//	O⊥	T//	T//
Melting point/°C	15.0	35.0	46.5	—	—	—
Long spacing/nm	3.5	3.2	3.1	3.25	3.15	3.13
Short spacings/nm	0.42	0.42	0.46	0.421 (s)	0.536 (m)	0.538 (m)
		0.38	0.39	0.384 (s)	0.522 (w)	0.525 (w)
			0.38		0.493 (w)	0.522 (w)
					0.458 (s)	0.494 (w)
					0.437 (w)	0.462 (s)
					0.413 (w)	0.456 (s)
					0.391 (s)	0.441 (m)
					0.380 (s)	0.409 (w)
					0.364 (w)	0.386 (s)
						0.376 (s)
						0.362 (w)
						0.359 (w)

^1^ 001 long spacing and characteristic short spacings are determined for LLL β′ and β crystals occurring at specific temperatures on heating after melt crystallization (Figure 2a). Roman letters following short-spacing values mean relative peak intensity: s, strong; m, middle; and w, weak.

## Appendix A

ATR-FTIR spectroscopy employs an electric field, so-called evanescent wave, which is caused by the total reflection of the incident radiation at the interface between a sample and a high refractive index prism called internal reflection element (IRE) [13,27]. The evanescent wave generated in the sample surface region wanes as apart from the sample/IRE interface in the order of light wavelength. Therefore, only the chemical species in the surface region about a few μm thick cause IR absorption.

The characteristics of the evanescent wave significantly depend on the polarization of incident radiation. The s-polarized incident radiation generates an electric field parallel to the interface (// the y axis in Figure 4a and the p-polarized incident radiation generates an electric field containing both a parallel component (// the x axis) and a perpendicular component (// the z axis) [28]). As for a uniaxially oriented sample, i.e., isotropic in the xy plane, the absorption intensities with p and s polarizations, *A*_p_ and *A*_s_, are related to the in-plane (// the xy plane) and out-of-plane (// the z axis) extinction coefficients, *k*_//_ and *k*⊥, as follows in Equations (A1) and (A2):

*A*_p_ = *c*^A^ (*k*_//_ + *α k*_⊥_) (A1)

*A*_s_ = c^A^ (*β k*_//_ ) (A2)

Here, *c*^A^ is a constant, and *α* and *β* are parameters determined by incident angle *θ* and refractive indices of prism and sample, *n*_1_ and *n*_2_. In the present experimental conditions of *θ* = 45°, *n*_1_ = 2.4, and *n*_2_ = 1.5, *α* and *β* are estimated to be 6.3 and 3.7, respectively. Therefore, p-polarized spectra emphasize IR-active modes whose transition moments are in a direction about perpendicular to the IRE sampling face. s-polarized spectra emphasize IR-active modes whose transition moments are about parallel to the IRE face. Generally speaking, the z component of transition moments gives the most striking effect on polarized ATR spectra.
